# Potential Drug-Drug Interactions in Prescriptions to Patients over 45 Years of Age in Primary Care, Southern Brazil

**DOI:** 10.1371/journal.pone.0047062

**Published:** 2012-10-10

**Authors:** Jorge Juarez Vieira Teixeira, Márcia Terezinha Lonardoni Crozatti, Carlos Aparecido dos Santos, Nicolina Silvana Romano-Lieber

**Affiliations:** 1 Departamento de Análises Clínicas e Biomedicina, Centro de Ciências da Saúde, Universidade Estadual de Maringá, Maringá–Paraná, Brazil; 2 Departamento de Ciências Biológicas, Universidade Federal de São Paulo, Diadema-São Paulo, Brazil; 3 Departamento de Estatística, Centro de Ciências Exatas. Universidade Estadual de Maringá, Maringá–Paraná, Brazil; 4 Departamento de Prática de Saúde Pública, Faculdade de Saúde Pública, Universidade de São Paulo. São Paulo, Brazil; Federal University of Rio de Janeiro, Brazil

## Abstract

**Background:**

Few cross-sectional studies involving adults and elderly patients with major DDIs have been conducted in the primary care setting. The study aimed to investigate the prevalence of potential drug-drug interactions (DDIs) in patients treated in primary care.

**Methodology/Principal Findings:**

A cross-sectional study involving patients aged 45 years or older was conducted at 25 Basic Health Units in the city of Maringá (southern Brazil) from May to December 2010. The data were collected from prescriptions at the pharmacy of the health unit at the time of the delivery of medication to the patient. After delivery, the researcher checked the electronic medical records of the patient. A total of 827 patients were investigated (mean age: 64.1; mean number of medications: 4.4). DDIs were identified in the Micromedex® database. The prevalence of potential DDIs and major DDIs was 63.0% and 12.1%, respectively. In both the univariate and multivariate analyses, the number of drugs prescribed was significantly associated with potential DDIs, with an increasing risk from three to five drugs (OR = 4.74; 95% CI: 2.90–7.73) to six or more drugs (OR = 23.03; 95% CI: 10.42–50.91). Forty drugs accounted for 122 pairs of major DDIs, the most frequent of which involved simvastatin (23.8%), captopril/enalapril (16.4%) and fluoxetine (16.4%).

**Conclusions/Significance:**

This is the first large-scale study on primary care carried out in Latin America. Based on the findings, the estimated prevalence of potential DDIs was high, whereas clinically significant DDIs occurred in a smaller proportion. Exposing patients to a greater number of prescription drugs, especially three or more, proved to be a significant predictor of DDIs. Prescribers should be more aware of potential DDIs. Future studies should assess potential DDIs in primary care over a longer period of time.

## Introduction

Treatment with drugs is an essential tool of modern health care, but may also be a cause of illness and death, leading to a huge economic burden for society [1]. Although the concurrent use of multiple drugs often increases therapeutic effectiveness, certain combinations are harmful [2] and the growing use of new pharmacological agents has led to an increased risk of drug interactions [3]. Drug interactions are common in older adults [4] and usually result from shared metabolism pathways or intersecting drug action pathways [5]. The drugs most commonly implicated in major potential interactions are those used in the day-to-day clinical management of elderly patients with chronic diseases [6]. Given the wide variety of drugs prescribed [7], the safety of medications in primary care is a topic of considerable importance. Improvement in drug safety is essential in terms of patient morbidity/mortality as well as in economic terms [1]. Prescribers in all fields of medicine must become more aware of medication safety for older adults [8].

Studies on primary care report prevalence values for potential drug-drug interactions (DDIs) ranging 12% to 80% [3], [9]–[11] and prevalence values for clinically relevant DDIs ranging from 1.9% to 28.3% [2], [3], [9]–[13]. On the other hand, studies carried out in different healthcare settings report lower prevalence values for clinically relevant DDIs, ranging from 0.4% to 17.3% [14]–[24]. However, the studies cited differ with regard to patient characteristics, features of the interaction analysis software and sample size.

Few cross-sectional studies involving adults and elderly patients with major DDIs have been conducted in the primary care setting. Moreover, DDIs in ambulatory settings have not been widely studied [17]. In Brazil, a number of studies on clinically important potential DDIs have been carried out in different settings. Studies involving community-dwelling individuals report prevalence rates ranging from 4.6 to 17.6% [20], [24]. Studies conducted in hospital settings report prevalence rates ranging from 0.6 to 18.3% [23], [25]–[27] and a study carried out in a primary health care unit reports a prevalence rate of 6.8% [28]. To the best of our knowledge, this is the first large-scale study on primary care carried out in Latin America. The aim of the present study was to investigate the prevalence of potential drug-drug interactions in patients aged 45 years or older treated in the primary care setting in southern Brazil.

## Methods

### Setting and design

A cross-sectional observational study was conducted at all 25 Basic Health Units in the city of Maringá, state of Paraná, southern Brazil, from May to December 2010. The estimated population of the city is 357,117 inhabitants.

### Patients and data collection

A convenience sample involving 827 patients and their respective prescriptions was determined for the data collection. A researcher spent one day at each Basic Health Unit throughout the period of service (7∶30 am to 6 pm). Information was collected from prescriptions at the pharmacy of the health unit at the time of the delivery of medication to the patient. After delivery, the researcher checked the electronic medical records to retrieve all medications prescribed to the patient. All prescription drugs are provided free by municipal healthcare service, except indapamide, meloxicam, norfloxacin and rosuvastatin, which are not included on the list of standard public health drugs. Prescription drugs, age and gender were the variables of interest. All information was recorded on a standardized form. The criteria for the inclusion of patients were having been treated in primary care by the physician of the Basic Health Unit, age 45 years or older and the use of at least two drugs prescribed by a physician.

### Organization and data quality

Two methods were used to ensure greater accuracy of the findings. The data were first manually transferred to standardized forms, then double entered into the Epi Data 3.1® (The EpiData Association, Denmark, Europe) program and conferred patient by patient twice. The results between methods were compared for overall agreement.

### Analysis of potential DDIs

All potential drug-drug interactions were identified in the Micromedex® database (Thomson Reuters Inc., 2011. Micromedex Healthcare Series Greenwood Village/CO) according to the degree of severity (contraindicated, major, moderate, minor) and entered into the Epi Data 3.1® program. The interactions were also analyzed for potential risk. All potential DDIs were analyzed in pairs. When a pair of drugs was not available in the Micromedex® database, the interaction was not analyzed.

### Statistical analysis

The data were analyzed using the Statistica® program version 8.0 (StatSoft Company Information, Tulsa, Oklahoma, USA). In the univariate analyses of factors associated with potential drug-drug interactions, the chi-square test was used for the comparison of the groups. In the multivariate analysis, all variables with a p-value of <0.05 and all confounding variables were incorporated into the model. The data were controlled for gender, age and number of drugs. The odds ratio (OR) and respective confidence interval (CI) was calculated in the multivariate analysis for each variable. A p-value of <0.05 was considered statistically significant.

### Ethical considerations

This study received approval from the Ethics Committee on Research Involving Human Subjects, State University of Maringá, in accordance with Resolution 196/1996 of the Brazilian Ministry of Health. Patient consent was waived (document 119/2010) due to the fact that the study involved information from a secondary database. Moreover, the team of researchers did not have contact with the patients at any time during the study. The researchers complied with Resolution 196/1996, ensuring complete confidentiality and anonymity. The Center for Continuing Education and Training of Health Workers (CECAPS)/Municipal Department of Health also approved this study.

## Results

From May 1 to December 29, 2010, 827 patients aged 45 years or older were investigated at 25 Basic Health Units for the identification of potential drug-drug interactions. Most patients were between 45 and 69 years of age (70.1%; mean age: 64.1±10.6 years) and female (65.9%). The number of concomitant prescription medications ranged from 2 to 11 (mean: 4.4±1.8) and 43.1% of patients took more than four drugs. Among the 827 patients, 521 had a potential DDI, resulting in a prevalence value of 63.0%. More than three fifths (320/521) of these patients had one to two potential DDIs (mean 2.5±1.9). The prevalence of major DDIs was 12.1% (100/827) and among these 100 patients, 81.0% had at least one potential DDI (Table 1).

**Table 1:** General patients characteristics attendance in primary care.

Variable	Frequency (%)
**Gender**	
Female	545 (65.9)
Male	282 (34.1)
**Age (mean ± SD)**	64.1±10.6
45–59	298 (36.0)
60–69	282 (34.1)
70–79	172 (20.8)
≥80	75 (9.1)
**Drugs (mean ± SD)**	4.4±1.8
2	109 (13.2)
3–4	361 (43.7)
5–6	245 (29.6)
>7	112 (13.5)
**Number of DDIs** a **per patient (mean ± SD)**	2.5±1.9
1	203 (39.0)
2	117 (22.5)
3	96 (18.4)
4–5	64 (12.3)
6–14	41 (7.8)
**Number of major DDIs per patient (mean ± SD)**	1.2±0.5
1	81 (81.0)
2	16 (16.0)
3	3 (3.0)

a Drug-drug interaction.

No statistically significant differences were found regarding the presence/absence of potential DDI between men and women (p = 0.61) or different age groups (p>0.05). In both the univariate and multivariate analyses, the number of drugs prescribed was significantly associated with potential DDIs, with an increasing risk from three to five drugs (OR = 4.74; 95% CI: 2.90–7.73) to six or more drugs (OR = 23.03; 95% CI: 10.42–50.91) (Table 2).

**Table 2:** Predictors of potential drug-drug interactions in patients attendance in primary care (n = 827).

	Potential drug-drug interactions		Univariate analysis		Multivariate analysis^a^	
	Yes (%)	No (%)	OR (95% CI)	p value	OR (95% CI)	p value
Sex						
Male	181 (34.7)	101 (33.0)	1		1	
Female	340 (65.3)	205 (67.0)	1.08 (0.80–1.46)	0.61	1.10(0.78–1.41)	0.56
Age (Year)						
45–64	268 (51.5)	165 (53.9)	1		1	
65–69	94 (18.0)	53 (17.3)	1.09 (0.74–1.61)	0.66	1.05 (0.69–1.58)	0.55
70–74	66 (12.7)	40 (13.1)	1.02 (0.66–1.57)	0.94	1.00 (0.62–1.55)	0.84
75–79	44 (8.4)	22 (7.2)	1.23 (0.71–2.13)	0.46	1.26 (0.75–2.21)	0.29
≥80	49 (9.4)	26 (8.5)	1.16 (0.69–1.94)	0.57	1.11 (0.67–1.90)	0.53
Number of drugs						
2	27 (5.2)	82 (26.8)	1		1	
3–5	312 (59.9)	200 (65.4)	4.74 (2.90–7.73)	<0.001	4.84 (2.85–7.91)	<0.001
6 or more	182 (34.9)	24 (7.8)	23.03 (10.42–50.91)	<0.001	25.11 (9.98–48.63)	<0.001

a Adjusted for sex, age (year) and number of drugs. OR = odds ratio; CI = confidence interval.

A total of 160 active substances were prescribed to patients, 16 of which were not analyzed in the Micromedex® database, thereby enabling the assessment of 90%. Forty active substances accounted for 122 pairs of major potential DDIs, classified as Drug A and Drug B. The drugs of Group A most involved in major potential DDIs were simvastatin (23.8%), captopril/enalapril (16.4%) and fluoxetine (16.4%). Drugs acting on the cardiovascular system (CVS) accounted for 69.0% of potential major DDIs and those acting on the central nervous system (CNS) accounted for 28.5% (Table 3)

**Table 3:** Frequency of pairs of major potential drug-drug interactions in prescriptions dispensed in primary care (n = 122).

Drug A	Drug B	Frequency (%)	Potential risk
Amitriptyline,			
Nortriptyline	Clonidine	2 (1.6)	Decreased antihypertensive effectiveness
Atenolol	Clonidine	5 (4.1)	Increased risk of sinus bradycardia; exaggerated clonidine withdrawal response (acute hypertension).
Atenolol	Diltiazem	1 (0.8)	Increased risk of hypotension, bradycardia, AV conduction disturbances.
Bromazepam	Phenobarbital	1 (0.8)	Aadditive respiratory depression.
Captopril	Allopurinol	2 (1.6)	Hypersensitivity reactions (Stevens-Johnson syndrome, skin eruptions).
Captopril	Potassium	1 (0.8)	Hyperkalemia.
Captopril, Enalapril	Spironolactone	20 (16.5)	Hyperkalemia.
Lithium	Hydrochlorothiazide	1 (0.8)	Increased lithium concentrations and lithium toxicity (weakness, tremor, excessive thirst, confusion).
Lithium	Chlorpromazine	1 (0.8)	Weakness, dyskinesias, increased extrapyramidal symptoms, encephalopathy, and brain damage.
Cilostazol	Acetylsalicylicacid (AAS)	1 (0.8)	Increased risk of bleeding.
Clonidine	Propranolol, Verapamil	3 (2.5)	Increased incidence of sinus bradycardia.
Digoxin	Hydrochlorothiazide, Indapamide, Amiodarone, Spironolactone,	15 (12.3)	Digoxin toxicity (nausea, vomiting, cardiac arrhythmias).
Enalapril	Allopurinol	2 (1.6)	Hypersensitivity reactions (Stevens-Johnson syndrome, skin eruptions, anaphylactic coronary spasm).
Fluoxetine	AAS, Meloxicam, Diclofenac, Ibuprofen	20 (16.5)	Increased risk of bleeding
Fluoxetine	Fluconazole	1 (0.8)	Increased risk of cardiotoxicity (QT prolongation, torsades de pointes, cardiac arrest).
Fluoxetine	Imipramine, Nortriptyline	2 (1.6)	Tricyclic antidepressant toxicity (dry mouth, urinary retention, sedation) and an increased risk of cardiotoxicity (QT prolongation, torsades de pointes, cardiac arrest).
Fluoxetine	Haloperidol	1 (0.8)	Haloperidol toxicity (pseudoparkinsonism, akathisia, tongue stiffness) and an increased risk of cardiotoxicity (QT prolongation, torsades de pointes, cardiac arrest).
Ginkgo Biloba	Ibuprofen	2 (1.6)	Increased risk of bleeding.
Haloperidol	Amitryptiline, Imipramine, Chlorpromazine	4 (3.3)	Increased risk of cardiotoxicity (QT prolongation, torsades de pointes, cardiac arrest).
Insulin (NPH), Metformin	Ciprofloxacin, Norfloxacin	3 (2.5)	Changes in blood glucose and increased risk of hypoglycemia or hyperglycemia.
Propranolol	Haloperidol	1 (0.8)	Increased risk of hypotension and cardiac arrest.
Rosuvastatin	Ciprofibrate	1 (0.8)	Increased risk of myopathy or rhabdomyolysis
Simvastatin	Amiodarone, Ciprofibrate, Ciprofloxacin, Diltiazem, Fluconazole	29 (23.9)	Increased risk of myopathy or rhabdomyolysis.
Simvastatin	Warfarin	1 (0.8)	Increased risk of bleeding and an increased risk of rhabdomyolysis.
Sotalol	Furosemide, Sulfamethoxazole/Trimethoprim	2 (1.6)	Increased risk of cardiotoxicity (QT prolongation, torsades de pointes, cardiac arrest).

Table 4 displays data regarding the 521 patients with potential DDIs. For all age groups, potential moderate DDIs were the most frequent (70.2%), followed by potential major DDIs (19.2%). The age group with the highest frequency of potential major DDIs was 45 to 69 years (70.0%).

**Table 4:** Distribution de patients exposed to potential drug-drug interactions, attendance in primary care (n = 521).

Age	Number (%) of patients exposed to drug-drug interactions^a^			
	Major	Moderate	Minor	Total
45–59	37 (37.0)	134 (36.8)	19 (34.6)	193 (36.6)
60–69	35 (35.0)	118 (32.0)	22 (40.0)	176 (33.4)
70–79	18 (18.0)	79 (21.6)	10 (18.2)	108 (20.5)
80–94	10 (10.0)	35 (9.6)	4 (7.2)	50 (9.5)
All	100 (19.2)	366 (70.2)	55 (10.6)	521 (100.0)

a Patients exposed to more than one interaction were counted only one time, by their most relevant drug-drug interaction.

## Discussion

The mean age of the sample (64 years) was similar to that reported in other studies conducted on primary care (62 to 69 years) [2], [11]. The mean number of prescription drugs was 4.4, which is was consistent with a study conducted in similar settings in Italy (4.3 drugs) [29]. Approximately 43% of the patients were prescribed more than four drugs, which is similar to the frequencies reported in previous studies (30.4% to 50.5%) [11], [29]. Although the relative frequency in the present study is consistent with the two studies cited, it should be stressed that mean age and number prescription drugs were different.

The prevalence for all potential DDIs in patients treated in the primary care setting was 63%. Rates ranging from 9.2% to 78.8% have been described in patients under ambulatory care for all potential DDIs [11], [30]. This broad range of prevalence values may be partially explained by factors such as study design, methodology, definitions, characteristics of the population, number of medications prescribed, therapeutic traditions and compendium of drug interactions [10], [30], [31].

In the present study, the prevalence of potential major DDIs was 12.1% (100/827). This finding is in agreement with that described in studies involving ambulatory patients, which report values ranging from 3.8% to 16% [11], [12], [14], [29]. Population-based studies report prevalence values ranging from 10% to 17.6% [10], [20]. Major DDIs are considered clinically important and should be avoided by healthcare professionals, especially physicians and pharmacists. Pharmacists, in particular, should avoid dispensing combinations of drugs that may have serious DDIs [32]. In the present study, only 0.4% of the patients (3/827) had contraindicated DDIs, which is similar to figures reported in studies carried out in France (0.4%) [33] and Taiwan (0.2%) [18]. A previous population-based study conducted in Brazil using the same database found a frequency of 2.4% [20]. Although this result is much higher than the frequency found in the present study, the difference may partially be explained by the study design and characteristics of the population.

No statistically significant differences were found between men and women regarding DDIs, which is consistent with findings described in previous studies carried out in primary care settings [11], [29], [34] as well as investigations carried out in other settings [35], [36]. Age is another important variable to consider in relation to drug use. In the present study, no statistically significant independent association was found regarding age, which is in agreement with a number of studies conducted in different settings [20], [22], [37] and in disagreement with other studies that report a positive association between age and potential DDIs [11], [20], [29], [38].

Three or more drugs prescribed to patients was significantly associated with potential DDIs. Similar studies report a trend of increasing prevalence of potential DDIs with the increase in the number of drugs prescribed [11], [29], [33]. Research worldwide has shown that polypharmacy (5 or more drugs) [39] contributes to the increased risk of potential DDIs [18], [19] In the present study, approximately two fifths of the patients were taking five or more drugs. The increasing prevalence of potential interactions with age reflects the increase in the number of prescriptions [33]. The number of drugs prescribed and used by patients is a significant predictor of potential DDIs in pharmacoepidemiological studies. Thus, steps should be taken to promote rational therapy in primary care.

The three most active substances involved in potentially clinically significant interactions were simvastatin, captopril/enalapril and fluoxetine. Simvastatin, which is used to control elevated cholesterol levels, is one of the most widely prescribed drugs [40]. However, it requires considerable care and vigilance, as it may expose patients to an increased risk of myopathy or rhabdomyolysis [2], [16], [41]–[44]. Moreover, when used in combination with drugs with a potential DDI, the risk is even greater. An older age is a risk factor for rhabdomyolysis among statin users. Patients at high risk for developing rhabdomyolysis should be closely monitored for signs and symptoms of the disease [44]. Captopril/enalapril in combination with potassium-sparing drugs may increase the risk of hyperkalemia [15], [45]–[49]. Fluoxetine interacting with an anti-inflammatory agent may increase the risk of bleeding [21], [50]–[52]. The prescription of these drugs in combination should always be carefully analyzed according to risk/benefit ratio [7].

The largest number of active substances prescribed with major DDIs were related to the CVS (diuretics, ACE inhibitors, digoxin, beta-blockers and calcium channel blockers), which is similar to findings described in studies carried out in different settings [3], [19], [22]. An Italian study reports acetylsalicylic acid, digoxin and enalapril as the most prescribed substances [29]. A study carried out in Mexico City reports that drugs for the alimentary tract and metabolism were the most commonly prescribed, whereas drugs for diabetes accounted for only 6.7% versus 24.8% for drugs acting on the CVS. A higher frequency of prescription drugs acting on the CVS is expected among adults and elderly individuals. The second class of drugs most involved with major DDIs were those acting on the CNS, which is consistent with findings described in a previous study [33]. Public health services should have a standard list of drugs, respecting the epidemiological characteristics and actual situation of each health service. If prescribers have access to a wider array of drugs at the health service, they could prescribe safer drugs with less of a chance of potential DDIs.

The results of the present investigation are similar to those described in international studies that show an exponential growth in major DDIs. The prevention of clinically relevant interactions in primary care is the responsibility of all healthcare professionals. As treatment should offer the advantage of better pharmacotherapy for patients, it is imperative for physicians to be more aware of the risks involved when exposing patients to major DDIs. These predictable interactions are the primary responsibility of the prescriber rather than the patient and care must therefore be taken to develop reliable prescribing strategies that can be continuously monitored and revised [6]. A number of programs for analyzing potential drug-drug interactions and technical information are available. The collaboration of healthcare professionals regarding this problem could reduce the impact of potential DDIs on public health.

The present study has some limitations that should be addressed. First, the study involved a convenience sample and consisted of only one day of data collection at each Basic Health Unit. Second, the analysis of the pairs of potential drug-drug interactions was based on only one database. Three, the study was conducted in only one type of setting and the results may not be generalizable to other settings or patients in different age groups. Despite these limitations, the present study offers important information on the prescription of drugs with potential DDIs in patients aged 45 years or older in primary care.

In conclusion, the findings of the present study showed that the estimated prevalence of potential DDIs among adults and elderly individuals was high, whereas clinically significant DDIs occurred in a smaller proportion and within the rates reported in the literature. Exposing patients to a greater number of prescription drugs, three or more, proved to be a significant predictor of DDIs. Future studies should assess potential DDIs in primary care over a longer period of time.
